# Monte Carlo simulations and phantom modeling for spatial frequency domain imaging of surgical wound monitoring

**DOI:** 10.1117/1.JBO.28.12.126003

**Published:** 2023-12-14

**Authors:** Lai Zhang, Alistair Bounds, John Girkin

**Affiliations:** a Durham University, Department of Physics, Centre for Advanced Instrumentation, Durham, United Kingdom; b Occuity Ltd., Reading, United Kingdom

**Keywords:** surgical wound, spatial frequency domain imaging, biomedical imaging, Monte Carlo, photon diffusion

## Abstract

**Significance:**

Postoperative surgical wound infection is a serious problem around the globe, including in countries with advanced healthcare systems, and a method for early detection of infection is urgently required.

**Aim:**

We explore spatial frequency domain imaging (SFDI) for distinguishing changes in surgical wound healing based on the tissue scattering properties and surgical wound width measurements.

**Approach:**

A comprehensive numerical method is developed by applying a three-dimensional Monte Carlo simulation to a vertical heterogeneous wound model. The Monte Carlo simulation results are validated using resin phantom imaging experiments.

**Results:**

We report on the SFDI lateral resolution with varying reduced scattering value and wound width and discuss the partial volume effect at the sharp vertical boundaries present in a surgical incision. The detection sensitivity of this method is dependent on spatial frequency, wound reduced scattering coefficient, and wound width.

**Conclusions:**

We provide guidelines for future SFDI instrument design and explanation for the expected error in SFDI measurements.

## 1 Introduction

Surgical site infections (SSIs) are a common complication following surgical procedures and represent a significant burden for both the patient and national healthcare systems.^1^^,^^2^ The gold-standard approach for SSI diagnoses is based on the visual signs or symptoms at the wound site, for instance, the tenderness and reddening of the skin, which typically lags infection. This leads to a delay in the early detection and subsequent suitable and timely treatment of SSIs.^3^

Optical imaging tools can provide more detailed, accurate, potentially quantifiable, and earlier information on the wound healing process for detection, diagnosis, and monitoring of treatment, compared with visual inspection alone.^4^ Spatial frequency domain imaging (SFDI)^5^^,^^6^ is one promising method for such an improvement. It is a quantitative, wide-field, and noncontact imaging tool for mapping the optical properties of tissue with the ability to provide depth information.^7^ During the imaging process, three phases of sinusoidal spatially modulated illumination patterns are projected to the target tissue area and the diffused reflectance is imaged by a camera. Recorded images are demodulated to obtain the alternating component (AC) image $I_{\mathrm{AC}}$ for each spatial frequency as shown in Eq. (1),

$$I_{\mathrm{AC}}=\frac{\sqrt{2}}{3}\sqrt{{\left(I_{1}-I_{2}\right)}^{2}+{\left(I_{1}-I_{3}\right)}^{2}+{\left(I_{2}-I_{3}\right)}^{2}},$$ (1)

where $I_{1}$, $I_{2}$, and $I_{3}$ are a set of three-phase diffuse reflectance images for a single illumination spatial frequency. Following calibration, an inverse recovery method is used to obtain the reduced scattering and absorption maps from $I_{\mathrm{AC}}$.

SFDI has been characterized for a range of clinical applications, including assessment of burn wound severity at an early stage,^8^ predicting infection risk,^9^ and estimating burn depth^10^ in a preclinical model. Risk of diabetic foot ulcers^11^ and pressure ulcers^12^ has also been assessed, and SFDI has demonstrated the potential to detect early-stage dental caries^13^ by quantitatively identifying dematerialized areas of dental enamel and dentin.

To characterize the healing process and risk of SSI, the key is measuring the changes in the vertical structure (i.e., the path of the surgeon’s scalpel) via optical property maps for the wound.^14^ Laughney et al.^15^ designed phantoms with vertical inclusions varying the scattering coefficient and radius to help determine the SFDI resolution in breast tumor surgery. Wirth et al.^16^ assembled phantom blocks having laterally different optical properties to analyze the edge function on scattering and absorption maps. They noticed the edge response at the boundary of heterogeneity limiting SFDI’s lateral resolution but only conducted specific *ex vivo* tissue or phantom experiments without further modeling. The reason behind this is the partial volume effect^17^ due to discontinuity in the shape and optical properties^18^^–^^20^ in biological tissue. For our purpose, this impact at the heterogeneous interface needs to be closely modeled to better characterize the surgical wounds vertically.

In this paper, we perform an assessment of vertical heterogeneous surgical wounds through Monte Carlo modeling and by projecting sinusoidal patterns directly onto a model surgical wound. The edge response and detailed resolution of SFDI as an imaging method are analyzed via the $I_{\mathrm{AC}}$ in both simulation and phantom experiment. We applied a two-dimensional look-up-table (LUT) method to recover the reduced scattering map for the phantom to explore how the uncertainties propagate. Our results provide guidelines for future SFDI measurement in a range of clinical conditions.

## 2 Method

To model the propagation of light in biological media, there are two main approaches.^21^ One is the analytical method using the radiative transfer function, including diffusion equation approximations, and the other is the use of numerical methods, commonly Monte Carlo simulation. The analytical solution for the SFDI method is limited to a homogeneous tissue model, which could be layered as a series of planes. At the same time, the reduced scattering coefficient $\mu_{s}^{\prime }$ of the tissue should be much greater than the absorption coefficient $\mu_{a}$ ($\mu_{s}^{\prime }\gg \mu_{a}$), which is not feasible for characterizing a surgical wound. Here, we apply Monte Carlo simulations to aid with solving the diffuse reflectance of the surgical wound model in SFDI imaging.

### 2.1 Simulation

#### 2.1.1 Monte Carlo method

**Fig. 1:**
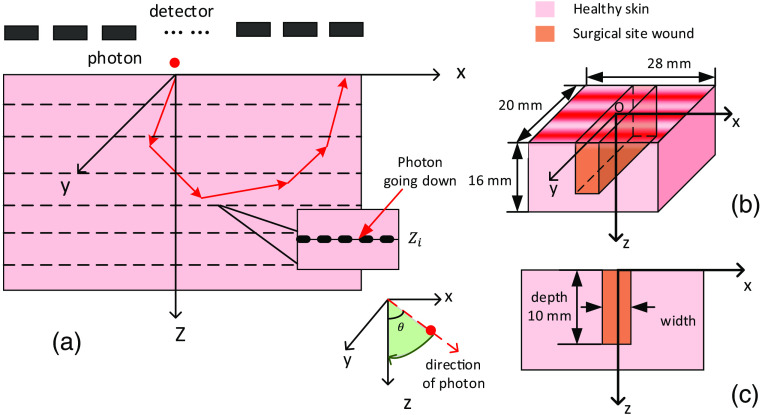
(a) The geometry of tracing the photon within the tissue. The example of the nondestructive detector at depth $z_{i}$ is shown. The direction of the photon going up and down is judged by the direction cosine between the direction vector and z axis. (b) The 3D structure of the surgical site wound. The collimated sinusoidal pattern (red colored) is projected to the tissue surface. (c) The xz section of the tissue.

To model the expected output of the practical diffuse reflectance $R_{d}$ of the wound, we modified the mcxyz^22^ (version July 22, 2019, downloaded on October 6, 2020) light transport modeling program to observe photon propagation in customized heterogeneous tissue. Figure 1(a) illustrates a typical photon path. Individual photons set off at the tissue surface with the same initial weight and direction. The tissue scattering properties cause a photon to potentially change propagation angle at each step while the absorption along the movement path reduces its weight. A sinusoidal illumination pattern is generated by the injected photon density following a sinusoidal probability distribution function along the y axis, such that the stripes are orthogonal to the wound long axis as shown in Fig. 1(b). When a photon escapes from the top surface, i.e., back scattered light, it is collected by the detector ending its journey as shown in Fig. 1. The minimum three-dimensional (3D) detection unit in simulation has a photon collection bin size of 0.02 mm in three dimensions. The illumination wavelength is originally set at 617 nm to match the experiment described in Sec. 2.2. This wavelength is selected to provide a suitable balance between scattering and depth penetration to epidermis and dermis.

To further monitor the photon migration in the tissue, we insert nondestructive detector planes at regular intervals 0.02 mm, to record the depth–dependent weight ${\text{Weight}}_{\text{down}}(x,y,z)$ within the tissue, as shown in Fig. 1, for the photon moving down through the tissue. When the photon moves to a new voxel, the weight of the photon will be updated and subsequently recorded by the detector planes. The upper facing detector will record the current weight of the photon travelling down through the tissue.

#### 2.1.2 Surgical wound model

During invasive surgery, the surgeon makes an incision through the patient’s skin to access the operation site. After the procedure, the incision will be sealed and form a wound with a vertical structure. We therefore built up a 3D tissue structure to mimic this as shown in Fig. 1(b) with its xz section in Fig. 1(c), where the wound is embedded in skin center with a cubic block structure and vertical boundary. During the wound healing process, we assume any wound infection or possible change only happens within the wound block. The wound width can change during the healing process, but the wound depth remains the constant. The skin remains homogeneous and uniform in optical properties.

As shown in Fig. 1, the tissue size is designed with tissue lengths of $l_{x}=28\;\mathrm{mm}$ (to eliminate the boundary effect), $l_{y}=20\;\mathrm{mm}$ (to include at least two periods of the sine pattern projection), and $l_{z}=16\;\mathrm{mm}$ while the depth of wound is 10 mm (to create a semi-infinite wound and skin structure^23^). We used 84,000,000 photons, the number being validated during preliminary simulations, to produce statistically repeatable results.

The absorption and scattering can both change during the wound healing process. The structural change typically results in scattering coefficient changes while absorption coefficient only changes the weight of the photon and is included via with Beer–Lambert law. As we are more interested in structural change, we assume the absorption coefficient of the wound remains constant and the scattering coefficient was varied for possible wound states. As the SFDI method can make assessment of scattering and absorption separately, we assign the wound and healthy skin a specific absorption coefficient $\mu_{a}$ and reduced scattering coefficient $\mu_{s}^{\prime }$. For healthy skin, we use typical human skin parameters^24^ at 617 nm, where the reduced scattering coefficient is $1.42\;{\mathrm{mm}}^{-1}$ and the absorption coefficient is $0.023\;{\mathrm{mm}}^{-1}$.^25^ The anisotropy, g, is fixed at 0.9 for both skin and wound.

#### 2.1.3 One-dimensional profile for wound monitoring

To simplify the observation of changes in wound width and the intensity of the AC image, here we define a one-dimensional (1D) AC profile. When looking at a small wound site area in our model, we can assume that the wound and skin region are each locally uniform with respect to their own optical properties. Therefore, we determine $I_{\text{curve},\mathrm{AC}}$ by averaging the intensity of the AC image $I_{\mathrm{AC}}$ along the y direction as in Eq. (2), where $N_{y}$ is the number of pixels in y direction. This helps to reduce the number of the photons that are launched into the original model as well as eliminating random noise in the Monte Carlo method.

$$I_{\text{curve},\mathrm{AC}}(x)=\frac{1}{N_{y}}\underset{y}{\sum }I_{\mathrm{AC}}(x,y).$$ (2)

### 2.2 Instrumentation

**Fig. 2:**
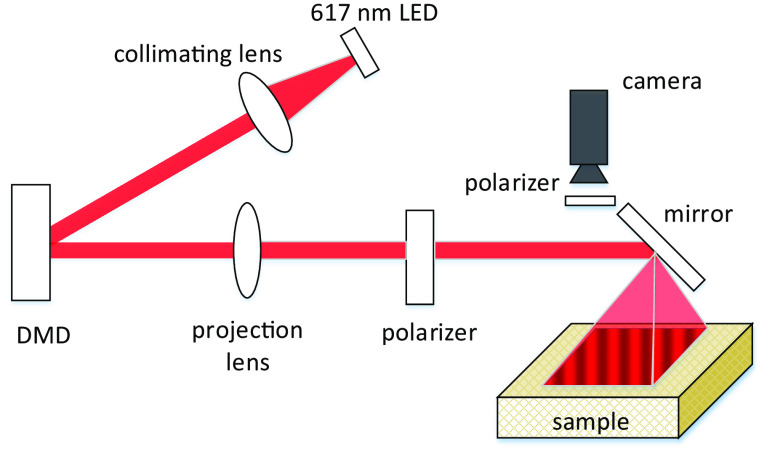
The geometry of the SFDI system.

We built a SFDI imaging system based on the OpenSFDI set-up configuration.^26^ A 617 nm LED (Thorlabs, M617D2) is projected onto a digital mirror device (Keynote Photonics, LC4500-NIR-EKT) to encode a sinusoidal illumination pattern onto the beam, which is then projected onto the sample. A USB camera (BFS-U3-13Y3M-C, Blackfly Camera, Edmund Scientific) with a 35 mm focal length lens was used to capture the diffuse reflectance image. The raw images are taken with a pixel effective size of 20  μm on the sample. The geometry of the SFDI system is shown in Fig. 2. Orthogonally aligned polarizers in the illumination path and in front of camera lens ensure that only back scattered light is imaged by rejecting surface reflections from the sample.

### 2.3 Phantom Design

A practical phantom is a significant element in the testing and calibration process. For the phantom base material, previous researchers had used agar,^23^^,^^27^ polydimethylsiloxane (PDMS),^28^^,^^29^ gelatin,^15^ and polyvinyl chloride-plastisol.^30^ An ideal phantom is supposed to be stable in both shape and optical properties to ensure no change over an extended time period either during imaging or storage.

**Fig. 3:**
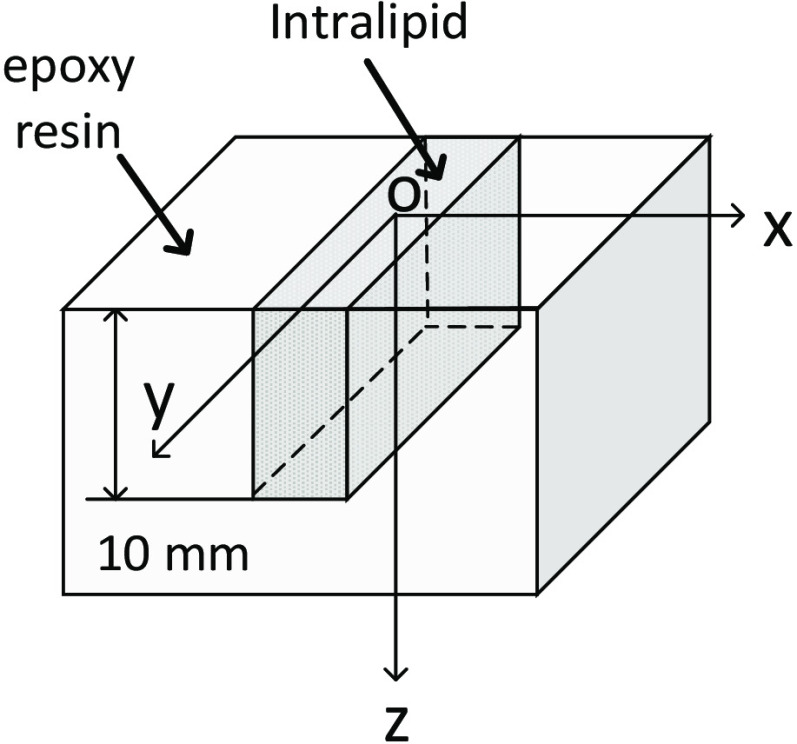
The geometry of the epoxy resin phantom.

To validate the simulation results, we used a clear epoxy resin to fabricate a cured polymer block and adding titanium oxide (${\mathrm{TiO}}_{2}$; Sigma-Aldrich) as a scattering agent. Extra absorbing materials were not added, but suitable materials could be incorporated assuming they are accurately controlled and uniform over the required volumes. The resin phantoms had a similar structure to the skin-wound imaging model, as shown in Fig. 3. The “wound” has the depth 10 mm filled with different concentrations of Intralipid solution (Sigma-Aldrich) to mimic different scattering with the wound. The absorption coefficient of the phantom was homogeneous as ${\mathrm{TiO}}_{2}$ and Intralipid does not absorb at our wavelength. The $\mu_{s}^{\prime }$ and $\mu_{a}$ of the resin “skin” and calibration sample were determined using the method used previous work.^31^ The $\mu_{s}^{\prime }$ of Intralipid was calculated using the Rayleigh–Gans approximation.^32^

## 3 Results

### 3.1 Simulation Results

#### 3.1.1 Accuracy in wound width determination

Here, we investigate the dependence of wound monitoring varying the wound reduced scattering coefficient $\mu_{s}^{\prime }$, wound width and illumination spatial frequency $f_{x}$. Five wound reduced scattering coefficients are considered based on the previous works^9^^,^^11^^,^^12^ (listed in Table 1), while $\mu_{a}$ is kept constant at $0.023\;{\mathrm{mm}}^{-1}$ and the anisotropy, g, is 0.9. The width values used were 0.5, 1, 1.5, 2, 4, and 6 mm and spatial frequencies $f_{x}$ of 0.1, 0.2, and $0.3\;{\mathrm{mm}}^{-1}$.

**Table 1:** Optical properties of five types of wound.

Tissue type	Reduced scattering ($\mu_{s}^{\prime }/{\mathrm{mm}}^{-1}$)
Very low scattering wound	0.47
Low scattering wound	0.71
No wound (healthy skin)	1.42
High scattering wound	2.84
Very high scattering wound	4.73

**Fig. 4:**
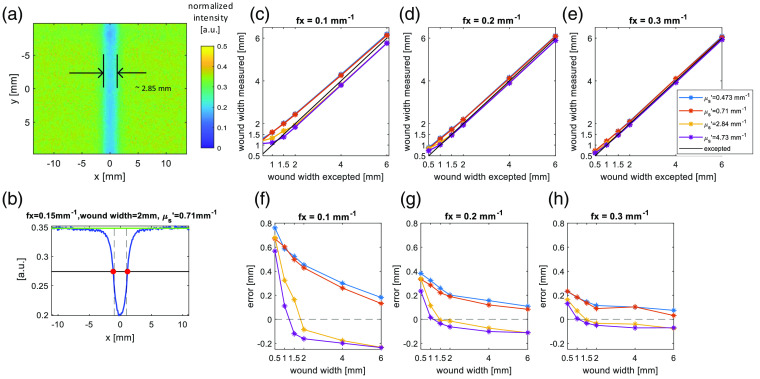
(a) The $I_{\mathrm{AC}}$ image of 2 mm width wound model with wound $\mu_{s}^{\prime }=0.71\;{\mathrm{mm}}^{-1}$. (b) The $I_{\text{curve},\mathrm{AC}}$ of the wound model in panel (a). The green line marks the skin intensity level and the black line mark the half maximum of the wound area. The vertical dashed line is the real wound area according to the wound model. (c)–(e) The measured wound width versus the real wound width. (f)– (h) The corresponding error of the wound width measurement for the data in panels (c)– (e). The dashed line is y=0.

The simulations are run for three phases of the light pattern and the AC images are calculated with Eq. (1). A typical simulation result is shown in Fig. 4(a), where the AC image result is color coded for intensity. The wound area, with its lower reduced scattering coefficient therefore lower returned intensity, can clearly be seen as the blue stripe in the center. Measuring the width of the blue region with ImageJ, we obtain the width value 2.85 mm in Fig. 4(a) demonstrating the partial volume effect.

Figure 4(b) illustrates the average intensity profile $I_{\text{curve},\mathrm{AC}}$ through the wound. The edge response in the $I_{\text{curve},\mathrm{AC}}$ spills out the shape of the wound. Thus, we used the full width half maximum value, which has the overall best estimation to determine the wound width from the simulations. Figures 4(c)–4(e) show the measured wound width versus the true wound width and Figs. 4(f)–4(h) plot the error of measurements there using error=result−truth. For the narrow wounds (≤1.5  mm), the width is significantly overestimated. The accuracy of the measurement increases with wider wound width, higher scattering coefficient, and higher spatial frequency (this matches the conclusion from Bassi et al.^33^). The wound width results from 1 to 2 mm wound model have unexpectedly low error in higher scattering wound models. The half maximum here is excellent for the wound width estimation.

**Fig. 5:**
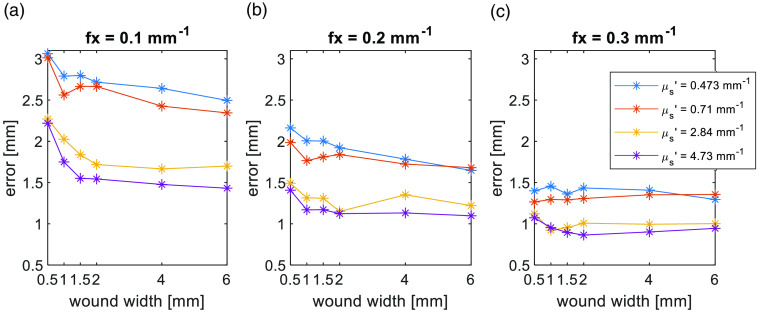
(a)–(c) The error of the $I_{\text{curve},\mathrm{AC}}$ measured at full width 90% maximum.

To explore whether edge response “spill out” affecting the wound area intensity, in Fig. 5, we plot the calculated wound width measurement error at the full width of 90% maximum with error=result−truth. SFDI results exaggerate the wound width as all the error values are positive. The edge response reduces with the higher scattering, higher spatial frequency, and wider width wound, leading to better accuracy in wound structure matching Figs. 4(c)–4(e). We further investigate this with the photon behavior around the skin wound interface in Sec. 3.2.1.

### 3.2 Phantom Experiments

To validate the simulation results, we use the SFDI system to image the mimicking phantom. The resin phantom had 2, 4, and 8 mm embedded “wound” to match the simulation design. The $\mu_{s}^{\prime }$ of the “skin” and “wound” is listed in Table 2. The $\mu_{a}$ of both resin and Intralipid solution is $0.002\;{\mathrm{mm}}^{-1}$. The spatial frequencies used in the experiment are 0, 0.1, 0.2, and $0.3\;{\mathrm{mm}}^{-1}$. The SFDI system is calibrated with a homogeneous resin sample of $\mu_{s}^{\prime }=1.2\;{\mathrm{mm}}^{-1}$ and $\mu_{a}=0.004\;{\mathrm{mm}}^{-1}$. The raw images are binned by 5×5 window before processing to improve the signal-to-noise ratio and speed up the image processing without any loss of useful resolution. The appSFDI^26^ code is utilized to recover the $\mu_{s}^{\prime }$ map.

**Table 2:** The $\mu_{s}^{\prime }$ of the phantom.

Tissue type	Reduced scattering ($\mu_{s}^{\prime }/{\mathrm{mm}}^{-1}$)
Skin	2.62
0.5% (v/v) Intralipid	0.53
1% (v/v) Intralipid	1.01
2.8% (v/v) Intralipid	2.21
3.5% (v/v) Intralipid	2.84
4.5% (v/v) Intralipid	3.35
5% (v/v) Intralipid	3.56

**Fig. 6:**
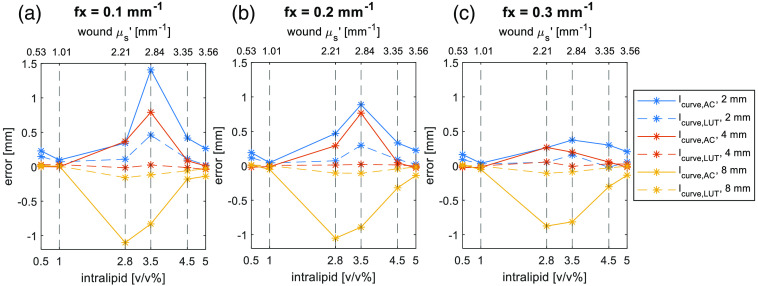
Comparison for the of wound width measurement error from $I_{\text{curve},\mathrm{AC}}$ and $\mu_{s}^{\prime }(x)$ by full width half maximum. (a) Spatial frequency $f_{x}$ is $0.1\;{\mathrm{mm}}^{-1}$. (b) Spatial frequency $f_{x}$ is $0.2\;{\mathrm{mm}}^{-1}$. (c) Spatial frequency $f_{x}$ is $0.3\;{\mathrm{mm}}^{-1}$. The solid lines are the results from the AC image while the dashed lines from the reduced scattering map.

Similarly to creating a 1D profile for the wound via $I_{\text{curve},\mathrm{AC}}$, we calculate $\mu_{s}^{\prime }(x)$ from

$$\mu_{s}^{\prime }(x)=\frac{1}{N_{y}}\underset{y}{\sum }\mu_{s}^{\prime }(x,y),$$ (3)

where the $\mu_{s}^{\prime }(x,y)$ is the recovered $\mu_{s}^{\prime }$ map image. The wound width is measured using the full width half maximum and the measurement error for both the $I_{\text{curve},\mathrm{AC}}$ and $\mu_{s}^{\prime }(x)$ is compared in Fig. 6. Looking at the $I_{\text{curve},\mathrm{AC}}$ (solid lines) in the plots, with the spatial frequency and wound width increasing, the measurement error decreases matching the simulation results. It should be noted that the error of 8 mm wound model is negative as the edge response reduces in wider wound.

There is a vaguely inverted U-shape trend in solid lines via the Intralipid concentration. The peak is around a concentration of 3.5% with the greatest uncertainty. This concentration provides a value of $\mu_{s}^{\prime }$ of $2.84\;{\mathrm{mm}}^{-1}$, very close to skin $\mu_{s}^{\prime }$ at $2.62\;{\mathrm{mm}}^{-1}$ leading to an $I_{\text{curve},\mathrm{AC}}$ with a flatter shape introducing a greater error from calculation. However, this does not constitute a downside in monitoring the wound with SFDI as the healed wound’s $\mu_{s}^{\prime }$ value approaches the $\mu_{s}^{\prime }$ value of the surrounding tissue. From another perspective, when wound complications appear where wound $\mu_{s}^{\prime }$ deviates further from the healthy skin $\mu_{s}^{\prime }$, the wound width can be measured with greater accuracy.

Comparing with the $I_{\text{curve},\mathrm{AC}}$ measurement, the error is significantly less in the $\mu_{s}^{\prime }(x)$ results. The calibration and LUT method effectively remove the modulation transfer function of illumination and imaging system. However, LUT method are not always predictable as the LUT is not linear^5^ and the accuracy of interpolation depends on the choose of the spatial frequency and the optical properties range.^34^

#### 3.2.1 Edge response

**Fig. 7:**
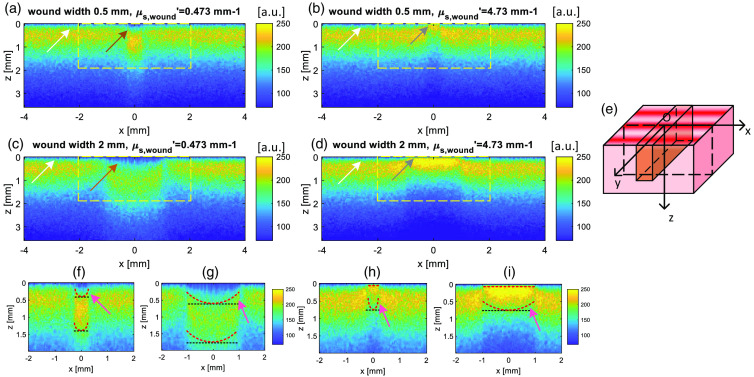
(a)–(d) The 3D edge response observed from the xz section of ${\text{Weight}}_{\text{down}}$ profile for the wound width 0.5 and 2 mm wound at $\mu_{s,\text{wound}}^{\prime }=0.473\;{\mathrm{mm}}^{-1}$ and $\mu_{s,\text{wound}}^{\prime }=4.73\;{\mathrm{mm}}^{-1}$. (e) The slicing method through xz section at y=0  mm. (g)–(i) The selected area in plots (a)–(d) shown with the dashed yellow rectangle. The red dashed contour line locates where the intensity is 70% of the maximum intensity within the whole wound area. The ideal 70% intensity contour line assuming the wound is infinite homogeneous and with same reduced scattering value is indicated with a black dashed line.

From the simulation and phantom experiments, we learn the spatial frequency, reduced scattering and wound width contribute to the SFDI lateral resolution. Here, we look closely into the photon behavior at the vertical interface between the healthy tissue and wound to understand influence on the transition to the images and width estimation. Four signature wound models with two wound widths 0.5 and 2 mm are selected with wound reduced scattering $\mu_{s}^{\prime }=0.473\;{\mathrm{mm}}^{-1}$ and $\mu_{s}^{\prime }=4.73\;{\mathrm{mm}}^{-1}$ from the simulation. The spatial frequency used is $f_{x}=0.1\;{\mathrm{mm}}^{-1}$ for four models. This serial of wound model combines the narrow and wide wound with the low and high scattering wound. We slice the ${\text{Weight}}_{\text{down}}$ through xz section at y=0 [see Fig. 7(e)] to view how the edge response contributes to the diffuse reflectance. In the intensity plots, we only note photons once they have undergone one scattering event.

In the low scattering wounds, as shown in Figs. 7(a) and 7(c), the brown arrows point out the first scattering event occurrences at a significant depth into the tissue. In the skin, the first scattering event occurrence is closer to the top surface noted by the white arrows. For the high scattering wounds, the first scattering event occurs at a shallower depth than in the relatively lower scattering healthy skin, as illustrated by the grey arrows in Figs. 7(b) and 7(d). As expected, photons penetrate more deeply in the lower scattering tissue. However, there are clear contributions to the profile from the tissue adjacent to the wound, which have different scattering values.

To investigate this edge response more closely, we plot the contour line of 70% maximum intensity. If we assume there is an infinite homogeneous wound area, any intensity contour line should be flat. However, all of the contour lines here show a U-shape indicating the influence of the tissue transition at the edge to the photon propagation. For the low scattering wound model as shown in Figs. 7(f) and 7(g), the photons propagate down further in the 2 mm wound than the 0.5 mm wound as the two contour lines are both deeper for 2 mm wound. Though the absorption reduces the “weight” of the photons when they travel further down, the wider wound still has significantly more photons at a greater depth (z≥1.5  mm). The photons between the real and ideal contour line as illustrated by the pink arrow in Figs. 7(f) and 7(g) are the photons entering the wound from the skin. They have less chance of being scattered back to the skin so generally travel within the wound resulting in the upper U-shape 70% contour line. There is decreased ${\text{Weight}}_{\text{down}}$ intensity in the healthy tissue adjacent to the wound, where the photons have been lost. The photons scattered into the wound from skin have already travelled longer distance from the skin area to the wound area. The photons moving from skin to wound have less “weight” than the “local” photons originally launched into the wound. They are not able to travel downward as far as the “local” photon, forming the lower U-shape 70% contour curve.

Inversely, in the high scattering wound [see Figs. 7(h) and 7(i)], photon’s first scattering event happens near the surface leading to a nearly flat 70% contour curve approximately at the surface for all the wound widths. Photons leave from wound to skin leading to the higher intensity in the skin area where adjacent to the wound. Similarly, the photons entering the healthy tissue, relative lower reduced scattering media, are less likely to be scattered back. Therefore the intensity is very low between the real and ideal 70% contour line as indicated by the pink arrow in Figs. 7(h) and 7(i), where photons are lost from wound to the healthy skin. Both pair of narrow and wide wound with same wound scattering properties show the influence of edge response is greater in narrower wounds. The high-scattering narrow wound area demonstrates lower intensity in diffuse reflectance than the wider wound, whereas the low-scattering narrow wound shows greater intensity.

**Fig. 8:**
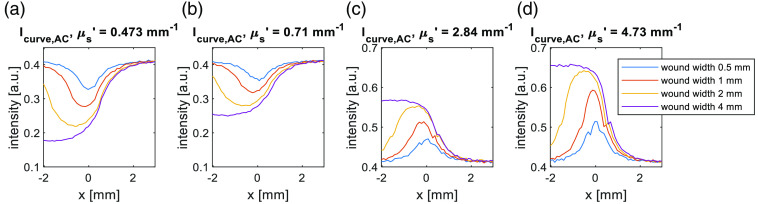
(a)–(d) Four wound width 0.5, 1, 2, and 4 mm $I_{\text{curve},\mathrm{AC}}$ are plotted particularly at the range where the curve approaching from the skin level to the centre of the wound area. The $I_{\text{curve},\mathrm{AC}}$ are aligned at full width 90% maximum by the 0.5 mm wound at right side.

To verify what we find from the ${\text{Weight}}_{\text{down}}$, we align the $I_{\text{curve},\mathrm{AC}}$ (spatial frequency used is $0.1\;{\mathrm{mm}}^{-1}$) to the full width 90% maximum to compare the transition around the wound-skin boundary area. From Fig. 8, the 0.5 and 1 mm wound curves are separated from other curves due to the more significant edge response and photons staying in the lower scattering media. The 2 and 4 mm wound curves almost overlap at the transition area. Thus, the relative contribution of the edge response effect decreases with the wound width increase matching the ${\text{Weight}}_{\text{down}}$ profiles.

## 4 Discussion and Conclusion

In this paper, we have explored SFDI’s capability to characterize the change in a vertical heterogeneous wound model. The 3D Monte Carlo method was applied to obtain a numerical solution by solving the diffuse reflectance and photon trajectory. We find SFDI’s lateral resolution on heterogeneous tissue is dependent on the reduced scattering, spatial frequency, and the wound width. The wound width itself contributes to the edge response, which influences the measurement error. The edge response at the skin-wound boundary is the main reason of the lateral error. The resin phantom experiment is in excellent agreement with the simulation results. We find:

**SFDI overestimates the wound width** due to the edge response at the wound-skin boundary. This can be compensated in the width measurement by selecting the certain intensity level of full width maximum, for example full width half maximum in our case.

**Trade-off (a): the sensitivity of detecting the change in the structure and reduced scattering coefficient.** From the previous work^5^ when the absorption is kept constant, the diffuse reflectance is able to better distinguish the change in the low scattering region. Here, we find the structure is easier to determine when the wound reduced scattering coefficient value is high. Therefore, the wound width measurement and the $\mu_{s}^{\prime }$ change cannot be sensitive to both properties simultaneously.

**Trade-off (b): the sensitivity of structure and the penetration depth.** One should select the spatial frequency and wavelength of the light source carefully. For the SFDI instrumentation parameter choice, one could make the spatial frequencies higher. As long as detection is possible from the required depth of tissue, this will help improve the measurement accuracy. One can use a longer wavelength, which will increase the depth of the detection resulting in a lower reduced scattering value, leading to a loss of structure sensitivity.

**SFDI is suitable for surgical wound monitoring, where the wound is embedded vertically in the skin.** The resin experiment matches the simulation results in showing the width change from both AC images and LUT results. In clinical practice, changes in the wound width and $\mu_{s}^{\prime }$ are more crucial than their exact value.

In this paper, we concentrate on varying $\mu_{s}^{\prime }$ and wound widths measurement assuming the absorption is constant. In a real wound situation, the absorption can vary in different wound stages. One can apply dual-wavelength measurement to better separate the absorption and scattering coefficients. In the future, we aim to image real surgical wound with SFDI and collaborating with clinicians to assess the wound condition. The 3D Monte Carlo simulation will be carried on for a two-layer surgical wound structure. Our numerical method can also be applied to monitoring the photon movement within the biological tissue with any structure.

## 5 Appendix: Recipe for the Resin Phantom

The healthy tissue part of the phantom was fabricated with epoxy casting resin and hardener (Glasscast50) in a ratio 2:1 by volume. Titanium dioxide was added to the mixture as a scattering agent (Sigma-Aldrich, 677469), allowing the phantom scattering to be precisely controlled based on the ${\mathrm{TiO}}_{2}$ concentration. The “wound” depth was fixed to 10 mm while the wound width varied in different models. The “wound” volume was filled with Intralipid emulsion (Sigma-Aldrich, 20 emulsion) at various concentrations to create different scattering coefficients.

## Data Availability

All relevant code and data are available from the authors upon reasonable request. Correspondence and requests should be addressed to the corresponding author.
